# Acupressure for persistent cancer-related fatigue in breast cancer survivors (AcuCrft): a study protocol for a randomized controlled trial

**DOI:** 10.1186/1472-6882-12-132

**Published:** 2012-08-21

**Authors:** Suzanna Maria Zick, Gwen Karilyn Wyatt, Susan Lynn Murphy, J Todd Arnedt, Ananda Sen, Richard Edmund Harris

**Affiliations:** 1 Department Family Medicine, University of Michigan, 24 Frank Lloyd Wright Drive, Ann Arbor, MI 48105, USA; 2 Michigan State University, B515 W Fee Hall, East Lansing, MI 48824, USA; 3 Institute of Gerontology, University of Michigan, 300 North Ingals, Ann Arbor, MI 48104-2007, USA; 4 VA Ann Arbor Health Care System, GRECC, Ann Arbor, MI 48104-2007, USA; 5 Psychiatry, University of Michigan, Rachel UpJohn Building, Ann Arbor, MI 48103, USA; 6 Department of Family Medicine, Department of Biostatistics, University of Michigan, 1018 Fuller Street, Ann Arbor, MI 48104-1213, USA; 7 Department of Anesthesiology, University of Michigan, 24 Frank Lloyd Wright Drive, Ann Arbor, MI 48105, USA

**Keywords:** Acupressure, Persistent fatigue, Breast cancer, Cancer survivor, Sleep quality, Ecological momentary assessment, Traditional Chinese Medicine, Actigraphy

## Abstract

**Background:**

Despite high levels of clinically significant persistent cancer related fatigue in breast cancer survivors few treatments are currently available and most pose a significant burden on the part of the woman. Acupressure, a component of Traditional Chinese Medicine, has been shown to decrease fatigue levels by as much as 70% in cancer survivors while being inexpensive, non-toxic and an easy to use intervention. The primary aim of this study was to determine the efficacy of two types of self-administered acupressure (relaxation acupressure and stimulating acupressure), compared to standard of care on fatigue severity. Secondary aims were to evaluate the efficacy of two types of acupressure on sleep and kinetic parameters required for implementation of acupressure in a clinical setting; The purpose of this paper is to share the methodology used including challenges and insights.

**Methods/design:**

This study is a three group, randomized clinical trial. 375 breast cancer survivors at least 12 months after completion of cancer treatments, with moderate to severe persistent fatigue, are being randomized to one of 3 groups: self-administered relaxation acupressure; self-administered stimulating acupressure; or standard of care. Participants are assessed at baseline, 3 weeks, and 6 weeks followed by a 4-week follow-up period. The primary aim is to examine the effect of 6-weeks of relaxation acupressure compared to stimulatory acupressure or standard of care on fatigue as assessed by: weekly self-report using the Brief Fatigue Inventory; objective daytime physical activity on actigraph; or fatigue patterns assessed 4-times daily using a visual analog scale. Secondary endpoints include depression, anxiety, self-efficacy, and sleep quality.

**Discussion:**

This study has the potential to develop a low-cost, self-care intervention for the most troubling of late-term effects in breast cancer populations, fatigue. The methods used may lend constructive ideas to other investigators working with this population and/or intervention.

**Trial registration:**

ClinicalTrial.Gov Trials Register NCT01281904

## Background

 There are nearly 3 million breast cancer survivors in the US [1]. Persistent cancer-related fatigue (PCRF) is one of the most distressing symptoms experienced by breast cancer (BC) survivors [2,3]. Rates of PCRF in BC survivors range from 30% to 82% and persist for as long as 10 years after diagnosis [3-7]. PCRF is associated with decreased quality of life, [5,6,8] and decreased sleep quality [5,6]. Beyond poor quality of life, subjective reports of low levels of fatigue, at diagnosis, in BC survivors predict longer recurrence-free and overall survival even after adjusting for key clinical and socio-demographic confounders [9]. Thus, decreasing the burden of PCRF could lead to a positive impact on women’s quality of life as well as improve their clinical status. Currently, however, there are few treatment options for PCRF.

Acupressure is a technique derived from acupuncture, a component of Traditional Chinese Medicine (TCM). In acupressure, physical pressure is applied to acupuncture points by the hand, elbow, or with various devices to treat disease. Acupressure has several advantages over other potential PCRF treatments: it is self-administered with little effort and time on the part of the patient; it is well tolerated; low-cost; and requires minimal instruction by clinic staff, e.g., nurse. Pilot randomized controlled trials (RCT) have demonstrated that self-administered acupressure can significantly decrease PCRF by as much as 70% from baseline levels in cancer survivors [10]. Acupressure can also have positive effects on sleep quality [11,12] and sleep quantity [10,12] in cancer patients and other chronically ill populations, but the degree to which these sleep improvements influence improvements in PCRF has not been explored. In a pilot clinical trial, Zick and colleagues [13] found that relaxation acupressure (RA) reduced PCRF more than stimulatory acupressure (SA), although PCRF was reduced by both treatments. In a separate study, Harris and colleagues [14] showed that sleepiness was increased to a greater degree following RA compared to SA. Based on these findings we designed a RCT to investigate the effect of self-administered acupressure on PCRF and sleep in a large group of breast cancer survivors. Below is an outline of our study design.

### Study aims and hypotheses

The hypothesis of this project is that self-administered RA will result in improvements in measures of PCRF, quality of life, and sleep quality, compared to SA and standard of care. To assess this hypothesis, our primary aim is to examine the effect of 6-weeks of RA compared to a regimen of SA or standard of care on fatigue as assessed by:

A. weekly self-report using the Brief Fatigue Inventory (BFI), our primary outcome.

B. objective daytime physical activity on a wrist worn accelerometer using average activity counts/minute.

C. fatigue severity assessed 4 times daily using a visual analog scale recorded on wrist-worn accelerometer.

D. quality of life as assessed by Long Term Quality of Life Instrument (LTQL).

Our secondary aims include investigating the effect of 6-weeks of RA compared to a regimen of SA or standard of care on sleep quality of BC survivors as assessed by:

A. monthly self-report using the Pittsburgh Sleep Quality Index (PSQI), our primary outcome.

B. daily sleep/wake diaries and nighttime actigraphy.

C. examining the relationships between improvements in sleep quality and improvements in quality of life for SA and RA, as assessed by LTQL.

Our other secondary aim is to assess activity parameters required for implementation of an acupressure treatment in a clinical setting including the time to onset of a clinically meaningful effect as well as the duration of effect following cessation of the intervention. These factors are currently unknown for acupressure.

## Methods/design

### Study design

**Figure 1:**
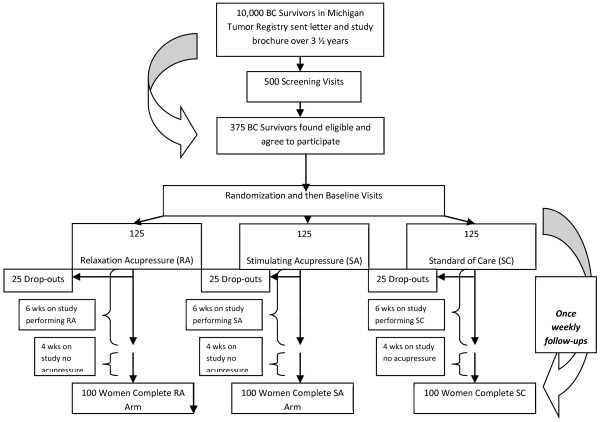
Flow of Participants Through the Study.

This is a randomized parallel group clinical trial with three study arms. The study schema and estimated recruitment numbers are presented in Figure 1. The study was approved by the University of Michigan Medical School Institutional Review Board and participants provided written informed consent.

### Study participants

The study participants are female breast cancer survivors (stage 0-IIIa), aged 18 years and older who were at least 12 months post-treatment and had a complaint of persistent, moderate to severe fatigue despite standard treatments. We detail eligibility criteria along with source of that material and the study personnel responsible for determining that criteria in Table 1. One of the principal investigators, who is a physician, reviews all eligibility documents and determines a woman’s suitability for participating in the study.

**Table 1:** Eligibility Criteria vs. Source Document

**Criteria**	**Source**	**Assessor**
Female breast cancer survivors	Medical Records, Michigan Tumor Registry, Medical History & Physical Exam	Study Nurse, Study Coordinator
Cancer free	Medical Records, Michigan Tumor Registry, Medical History & Physical Exam	Study Nurse, Study Coordinator
Completed treatment (i.e., surgery, chemotherapy, radiotherapy, immunotherapy, etc.) at least one year prior	Medical Records, Medical History & Physical Exam	Study Nurse, Study Coordinator
Capable of self-administering acupressure	Observation at screening visit	Study Nurse, Study Coordinator
Complaint of fatigue	Brief Fatigue Inventory score of ≥ 4	Study Nurse, Study Coordinator
Pregnant or planning to become pregnant	Pregnancy test, Medical History & Physical Exam	Study Nurse, Study Coordinator
Fatigue causing comorbidities (e.g. anemia, fibromyalgia diagnosis prior to breast cancer)	Medical Records, Medical History & Physical Exam	Study Nurse, Study Coordinator
Medication changes	Review of Concomitant Medications, Medical History & Physical Exam	Study Nurse, Study Coordinator
Acupuncture/Acupressure w/in 6 months	Communication with potential participant	Study Coordinator
Non-Breast cancer diagnosis w/in 10 years	Medical History & Physical Exam	Study Nurse
Medications for insomnia	Medical Records, Review of Concomitant Medications	Study Nurse, Study Coordinator
Untreated major depressive disorder and suicidal ideations	PRIME-MD (‘several days’ indicated on question 2i)	Study Nurse, Study Coordinator
Final, overall eligibility	Potential participant’s final review of eligibility	Principal Investigator

### Randomization, blinding and allocation concealment

This is a single-blinded study. Participants, investigators, study nurses and all study staff are blinded. However, the nurses who teach the acupressure will know if participants are assigned to the standard of care arm or one of the two acupressure arms. The randomization scheme was created by the study statistician. Within each county under study, eligible patients are being randomized into one of these three groups: RA, SA or standard of care in a 1:1:1 ratio for a target total of 375 patients.

### Recruitment and enrollment challenges

There are multiple steps to obtaining an adequate sample size for an RCT consisting of cancer survivors. They are often no longer in the formal care system and must be located via alternative means. In the acupressure study, participants are identified through the Michigan Tumor Registry (MTR), which is run by the Michigan Department of Community Health (MDCH). The registry collects information about cancer incidence, stage, and to a limited extent, the kinds of treatment that patients receive. These data are reported to the registry from oncology facilities statewide. The MTR also includes cancer case information received from the metropolitan Detroit Surveillance, Epidemiology, and End Results (SEER) registry. The Michigan Cancer Surveillance Program, which operates the Michigan Tumor Registry, is a member of the North American Association of Central Cancer Registries (NAACCR). The registry is certified by NAACCR as meeting all standards for quality, completeness, timeliness, and unresolved duplicate records (1 per 1000 tumors) and is estimated to be greater than 95% complete based on external audit findings.

The initial step is for the study team was to obtain Institutional Review Board (IRB) approval from the universities involved as well as the MTR. Next, the study team worked collaboratively with the staff at the MTR to clarify the population of interest. MTR Data were requested for women diagnosed between January 1st, 2006 and December 31st, 2010, from 5 counties in Michigan (Wayne, Washtenaw, Ingham, Genesee, and Kent). These counties represent significant population centers (Detroit, Ann Arbor, Lansing, Flint, and Grand Rapids), and areas of racial and ethnic diversity.

The next step in the process of contracting the target population was by regulation conducted by the MTR. The State health department is required to make the first contact according to State regulations, since the MTR was formed under the auspices of the State’s Public Health Authority. Specifically, the MTR first contacts the physicians who diagnosed and treated the patients in the surveillance registry. The letter sent to the physicians explains the purpose of the mailing, asks about any contraindications to contacting the patient, and requests updated contact and vital status information. It includes a response form with the letter. A passive response approach is used. This method provides physicians with MTR fax and phone numbers, and instructions to respond by a specific date, or the MTR will assume no contraindications to contacting the patient, and that she is alive and residing at the address provided.

Following the physician contact, the MTR staff notifies eligible women by mail of the opportunity to enroll in the Acupressure for Persistent Cancer Fatigue Study. This mailing asks if a member of the research team may contact them about this research. As with the mailing to physicians, a response form is included with the patient letters. The MTR staff also responds to calls from patients with inquiries. The recruitment project supervisor at MTR serves as a backup for handling the most difficult calls. Multiple attempts are made to reach the women, and up to 3 mailings are sent to BC survivors, with the last letter being sent by certified mail. Current addresses for mailings that are returned as undeliverable are tracked using online LexisNexis services.

The final step to recruitment for the MTR is when identifiable data is released to the study office on those women who gave their permission to be contacted about possible study enrollment.

From this point on, the research team approaches the women referred by the MTR. The research staff contacts the women (with up to six phone call or email attempts) to discuss enrollment and screening for the Acupressure for Persistent Cancer Fatigue Study.

### Multilevel screening for eligibility and consent procedures

#### Pre-screening

Upon receiving the release of contact information from the MTR, the interested study candidates are contacted by the study coordinator. The study coordinator explains the study to them and also asks the potential participant a short number of questions to confirm their potential eligibility and interest in the study.

#### Visit 1 - screening visit (to determine eligibility)

Once the pre-screening is completed and the study candidate appears eligible the study coordinator schedules a ‘screening visit’ with the study nurse in the Michigan State University (MSU) County Extension office closest to the potential participant’s home location in the 5 counties in Michigan: Wayne, Washtenaw, Ingham, Genesee and Kent.

At the screening visit Informed Consent is reviewed, signed and all questions are answered. A brief physical exam including vitals is performed. Socio-demographic information, medical history and a list of current medications are collected. The study nurse also conducts a blood draw for basic information on the women’s health status. Finally, potential participants complete a short battery of self-administered paper and pencil questionnaires, which help determine study eligibility (Table 1).

#### Pre-baseline

The PI reviews the documents and confirms the eligibility for the study candidate. An Actiwatch-Score (hereafter referred to as Actiwatch-S; Phillips Respironics, Bend OR), a wrist-worn accelerometer, and a study log book along with instructions are mailed to them. An Actiwatch-S is a wristwatch-like device that continuously measures activity levels and also allows for input of fatigue severity scores. The device is worn by the participant throughout the study period. The study log book accompanies the Actiwatch-S and has additional questions about activity and sleep quality to be completed within and across days in the study. The participants enter their fatigue scores in the log book as they do in the Actiwatch-S, which provides a cross-validation of the time-stamped fatigue entries. The participants are instructed to wear the Actiwatch-S for one week prior to the Baseline Visit and to record their fatigue three times a day at pre-scheduled times after hearing an audible alarm. Prior to the Baseline appointment, the participant is randomized into one of the three arms.

#### Visit 2- baseline visit (week 0)

At the visit participants fill out questionnaires as indicated in Table 2. If the participants are randomized into one of the acupressure treatment arms, they are given acupressure instructions by the study nurse. A DVD, a handout describing acupressure, and illustrations of the proposed acupressure points are also given to the participants. On this visit the participant returns the Actiwatch-S and the study logbook. The data from the Actiwatch-S are downloaded and the watch is reinitialized and given to the participant along with a new log book.

**Table 2:** Study Visits

	**Visit 1**	**Daily**	**Visit 2**	**Visit 3**	**Visit 4**	**Weekly Phone Calls**	**Visit 5**
	**Screening (within 60 days of baseline)**	**Days (−7 to 70)**	**Baseline Week 0**	**Week 3**	**Week 6**	**Week (−1, 1–2, 4–5 & 7–9)**	**Week 10**
Laboratory Values ^1^	X						
Medical Exam/History/Socio-demographics	X						
Urine Pregnancy Test ^1^	X						
PRIME-MD^2^	X						
HADS ^2^	X				X		X
BFI ^2^	X		X	X	X	X	X
Concomitant Medication & Supplements	X						
Sleep Diary	X				X		
Group Randomization ^3^			X				
Actiwatch Data Collected		X	X^4^	X^4^	X^4^		X^4^
LTQL			X		X		X
BSAPQ ^2^	X		X		X		X
GSE ^2^			X		X		X
BPI^2^	X		X		X		X
Study Logbook		X	X	X	X		X
PSQI ^2^	X				X		X
VAS for Pain ^2^	X						
Berlin Questionnaire	X						
Therapy Evaluation Questionnaire			X		X		
Perform Acupressure ^5^			X	X	X		
Assess Treatment Fidelity – Self efficacy Measure				X	X		
Adverse Events				X	X	X	X
Assessment of Blinding					X		

^1^ Complete Blood Count; blood for correlative tests, blood for future DNA analysis, Urine pregnancy test as appropriate.

^2^ HADS = Hospital Anxiety Depression Scale; BFI = Brief Fatigue Inventory; and PSQI = Pittsburgh Sleep Quality Index; LTQL = Long Term Quality of Life Instrument; BSAPQ = Breast and Surrounding Areas Pain Questionnaire; GSE = General Self Efficacy Scale; BPI = Brief Pain Inventory; VAS for Pain = Visual Analog; PRIME-MD = *Primary Care Evaluation of Mental Disorders.*

^3^ Participants are randomized to one of three treatments: Standard of Care; Relaxation Acupressure; or Stimulating Acupressure.

^4^ Data file downloaded at these visits.

^5^ Does not apply to standard of care arm.

#### Visit 3 (week 3)

The participant completes the BFI questionnaire and adverse events, if any, are noted. Participants are tested for the accuracy of locating their acupoints and the skill of precisely stimulating the points (acupressure fidelity measure), as appropriate. Participants return the Actiwatch-S and the log book. The data from the Actiwatch-S is downloaded; the watch is re-initialized, and returned to the participant along with a new study log book.

#### Visit 4 (week 6)

Marks the end of doing acupressure and the beginning of the follow-up phase: Participants complete a battery of questionnaires, are asked about any adverse events, assessed for blinding, and tested for acupressure fidelity (see Table 2). Data from the Actiwatch-S are downloaded and the reinitialized and a14-day sleep diary is given to them. If the participant belongs to the acupressure treatment arm of the study they are asked to stop acupressure treatment for the next 4 weeks.

#### Visit 5 - final (week 10)

Participants complete all questionnaires (Table 2.) and the 14-day sleep diary, study log book and Actiwatch-S are collected.

#### Weekly phone calls (weeks 110)

Participants are contacted once per week to administer the BFI questionnaire, ask about any medical concerns, change in medications, and any questions regarding sleep diary, study log book, acupressure or Actiwatch-S.

### Study measures

#### Subjective study measures

Below is a list of self-report measures that participants complete per the schedule in Table 2.

*Hospital Anxiety Depression Scale):* this 15-item questionnaire is a widely used instrument to measure depression and anxiety [15].

*Primary Care Evaluation of Mental Disorders (PRIME-MD):* It is a 26-item self-administered questionnaire that screens for five of the most common groups of psychiatric disorders in primary care: depression, anxiety, alcohol, somatoform and eating disorders [16].

*Brief Fatigue Inventory:* The BFI assesses the severity of fatigue and the impact of fatigue on daily functioning in patients with fatigue due to cancer and cancer treatment [17].

*Actiwatch-S to collect fatigue severity:* Four times daily participants indicate on a scale from 0 = “No Fatigue” to 10 = “Extremely Fatigued,” their fatigue severity at that moment. These scores are entered into the Actiwatch-S upon completion of their acupressure treatment and at three pre-scheduled times, once in the morning (9:00 AM), once in the afternoon (3:00 PM) and once in the evening (9:00 PM).

*Long Term Quality of Life Instrument:* The LTQL is a 34 items questionnaire that is self-administered for evaluating functional impairment and the perceived effect of that impairment on quality of life in breast cancer survivors [18].

*Breast and Surrounding Area Pain Questionnaire:* The BSAPQ is used for measuring pain in the area of the breast (defined as the breast, armpit, side of the body, or arm on the operated side) on either the operated breast or the area from which the breast was removed [19].

*Brief Pain Inventory:* The BPI is used to assess average clinical pain [20].

*General Self-Efficacy Scale:* The construct of Perceived Self-Efficacy reflects an optimistic self-belief that one can perform a novel or difficult task, or cope with adversity in various domains of human functioning [21].

*Pittsburgh Sleep Quality Index:* The PSQI evaluates sleep disturbance over the past month. [22]. *Sleep Diary:* Sleep diaries are simple, non-invasive, self-report measures that provide night-to-night information on sleep pattern, quality, and relevant daytime behavior.

*Visual Analog Scale for Pain Severity:* The VAS for Pain will be given at the screening visit. It contains only one question which asks about overall pain intensity within a one week period and ranges from no pain up to worst possible pain. Participants are asked to place a tick mark along a solid 10 cm line.

*Berlin Questionnaire:* The Berlin Questionnaire asks questions to determine the likely presence or absence of sleep apnea [23].

*Therapy Evaluation Questionnaire:* The Therapy Evaluation Questionnaire asks about the subject’s perception of the treatment on a 7-point scale ranging from “not at all” up to “very” [24].

*Adverse Events:* Toxicity are graded according to NCI Common Toxicity Criteria version 4.02 [25].

*Treatment Fidelity and Self-efficacy Measure:* The acupressure educator asks the participant to identify each of their acupoints and to stimulate 1 point on the educator. Participants are also asked to record how long they should stimulate acupoints and how frequently treatments should be administered.

*Assessment of Blinding:* At the week 6 visit participants are asked if they believed they received “the stimulating acupressure”, “the relaxing acupressure”, or “don’t know”.

*Logbooks:* A logbook is used for double data entry and to verify actigraph data (both movement and recording when acupressure was performed).

### Objective study measure

#### Actiwatch-S is used to collect daytime and nighttime physical activity and symptoms

This wrist-worn accelerometer monitors the occurrence and degree of motion and measures the number of movements that exceed 0.01 g gravitation force. Activity data are downloaded to computer and sleep/wake activity is estimated using Actiware^®^ – Sleep software. It is important to emphasize that actigraphy does not measure sleep per se, but a behavior that is highly correlated with sleep—level of activity. Sleep episodes, both intended and unintended, can be reliably identified when low activity occurs in the presence of other indicators of sleep, such as self-reported sleep [26-29]. Participants are instructed to wear the Actiwatch-S at all times. If the Actiwatch-S is removed, removal and replacement times are recorded in a daily log book. The software uses a validated algorithm to compute standard parameters of sleep/wake and activity cycles for each day of recording, which are checked by our study staff using the log books. The primary outcomes for nighttime actigraphy are total sleep time (total minutes scored as sleep by the Actiware^®^ algorithm) and sleep efficiency (total sleep time/time in bed*100,%).

**Figure 2:**
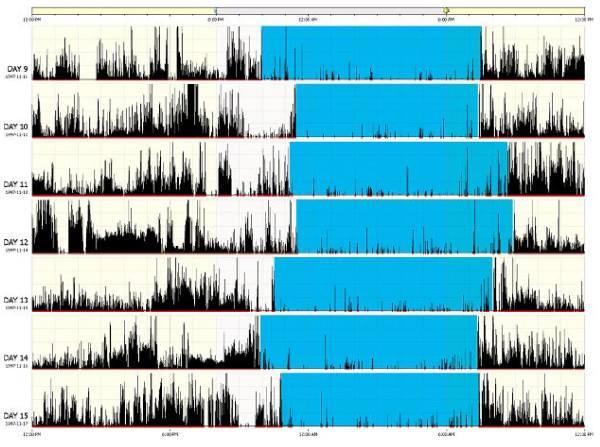
Representation of an Actigraph.

Actigraphy has also more recently been used by members of our research team to examine daytime physical activity by measuring activity patterns [28-32], the effects of a physical activity intervention [30], and to assess fatigue. The assessment of fatigue using this method improves upon existing recall-based methods of symptom reporting in which people tend to underreport or report only peak or recent symptoms [33] Figure 2.

## Acupressure intervention

The study utilizes individual instruction by acupressure educators to teach study participants about the acupressure intervention. Acupressure educators are local study staff (located in the county where the women are being recruited from the cancer registry) made up of nurses who have been trained by one of the study co-principal investigator (Co-PI), who is a professional acupuncture practitioner. The study Co-PI meets with the local acupressure educators for an initial training session. At this session he explains the basic philosophy of TCM and acupressure, demonstrates both sets of study acupressure points on himself and on the educator. He also gives the educators a DVD of a person performing the acupoints, laminated illustration of acupoints along with a written description of where the points are located. He also asks the educators to demonstrate the correct location of the acupoints on themselves as well as proper pressure and stimulation techniques.

The Co-PI makes a site visit to observe the acupressure educator performing both sets of acupoints (stimulating and relaxing) on themselves. He notes how many acupoints are correctly located and makes any needed corrections. He also asks them to apply the correct amount of pressure and stimulation on a point on him. The Co-PI has a checklist that interveners must match at a 95% rate. After the acupressure educator has enrolled two participants, the Co-PI returns to the site to check for the efficacy of the acupressure educators and then twice yearly afterwards. These assessments of the acupressure educators are how we measure their treatment fidelity. Acupressure educators can request additional clarification and instruction as needed.

From our previous study, participants can be taught to perform their acupoints within 15 minutes [13]. Acupressure educators meet with each participant separately and demonstrate the location of each acupoints on themselves and on the participants. Participants also have the acupoints marked on them with adhesive colored dots to reinforce the location of the acupoints for the first few days. At the end of the session participants are asked to demonstrate the location of all their acupoints to the educator and appropriate adjustments are made.

Acupressure educators also demonstrate on the participant the appropriate level of pressure to apply on the acupoints and the correct stimulating motion. Participants are then asked to stimulate points on the educator until it is determined that they are using the correct pressure and stimulation. Participants are told and given a handout with the following information for correctly performing their acupressure:

“Once you have identified the pressure point location, use the pad- not the tip- of your index finger to apply pressure. Place your index finger on the selected point and turn it in a clockwise direction. Apply firm pressure to the point using a light to moderate touch depending on your sensitivity. The points may feel somewhat tender to the touch, and pressure should be enough to illicit that sensation. There should be a distinct feeling around or at the site of pressure. This may be felt as numbness, warmth, tingling, aching or buzzing sensation. If no sensation is felt, try applying more pressure. Please note that some points are more sensitive than others, and in fact you may not feel any sensation at certain points."

"Pressure should be applied for **3** minutes per point. Treatments are done every day, **once** per day. Pressure points should be done in the order provided, starting from the head down to the feet. Apply pressure to **ONE** point at a time. If a point is located on both sides of the body, apply pressure to the **RIGHT** side, followed by the **LEFT** side prior to proceeding to the following point."

"Do **NOT** lose contact with the point. If you need to, you may take a break in between points for a couple of minutes. Please do **NOT** take a break in the middle of applying pressure to a point."

"Acupressure can be performed sitting, standing or lying down.”

Participants may return at any time to be retrained or ask clarifying questions.

Acupoints were chosen by consensus of four acupressure practitioners and based on a previous study design in students with sleep disturbances as well as TCM theory for treating insomnia and fatigue. Practitioners had all been in practice for at least 2 years actively seeing patients. They also had been trained as and received one of more of the following degrees; a Naturopathic Doctorate (ND), masters in TCM or Oriental Medicine and a license of acupuncture (L.Ac.) or a diploma in acupuncture (Dipl. Ac.). Practitioners were asked to choose a set of relaxing and stimulating acupressure points based on a Western diagnosis of fatigue that could be reasonably reached by participants, i.e., not the middle of the back, and not so many points that it would take an excessive amount of time to complete a treatment. Following is a description of the two acupressure treatments and the definition of standard of care:

### Relaxation acupressure

In addition to standard of care participants are asked to apply pressure on each of following points (bilaterally where indicated). There are five acupoints with four of the acupoints performed on both the left and the right sides of the body giving a total of nine points to stimulate. Each of the nine acupoints is stimulated for three minutes per point giving a total treatment time of 27 minutes done once daily. The acupoints are:

"***Yin tang*** (Unilaterally), ***Anmian*** (Right and Left/bilaterally), **Heart 7 (HT7)** (Right and Left/bilaterally), **Spleen 6 (SP6)** (Right and Left/bilaterally), **Liver 3 (LIV3)** (Right and Left/bilaterally)."

### Stimulating acupressure

In addition to standard of care participants are asked to apply pressure on each of following points (bilaterally where indicated). There are six acupoints with four of the acupoints performed on both the left and the right sides of the body giving a total of ten points to stimulate. Each of the ten acupoints is stimulated for three minutes per point giving a total treatment time of 30 minutes done once daily. The acupoints are:

"***Du 20*** (Unilaterally), **Conception Vessel 6 (CV6)**, **Large Intestine 4 (LI4)** (Right and Left/bilaterally), **Stomach 36 (ST36)** (Right and Left/bilaterally), **Spleen 6 (SP6)** (Right and Left/bilaterally), **Kidney 3 (K3)** (Right and Left/bilaterally)."

**Figure 3:**
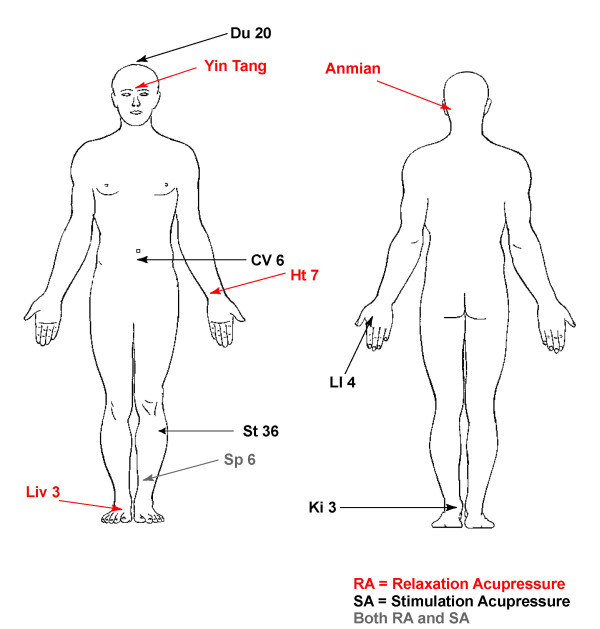
Acupressure Point Locations.

Locations of the acupoints can be found in Figure 3.

### Standard of care

Participants are asked to continue doing whatever their healthcare providers advise for PCRF. We anticipate that most women will be treated per the National Comprehensive Cancer Network (NCCN) Clinical Guidelines for Fatigue V1.2009 (NCCN Guideline for fatigue) [34]. These guidelines recommend stress management or cognitive behavioral therapy; short naps and possible use of stimulants and sleep medications. Participants will be asked to continue any current treatment and not to start or stop treatments over the course of the study. All treatments for fatigue will be recorded.

## Statistical issues

### Statistics and power analysis

Primary Aim: The primary aim of this study is to investigate the differential effects of different modes of acupressure on fatigue in breast cancer survivors. For this, we are focusing on three fatigue outcomes, namely, BFI, which will be administered weekly, a measure of average daily physical activity and a self-reported fatigue score, both based on Actiwatch-S data. A BFI is collected weekly and provides a measure of average degree of fatigue, a score that ranges between 0 and 10, with higher numbers indicating more fatigue. We will be analyzing the BFI outcome over phases. The first phase BFI is obtained by averaging week-1 to week-3 measures (mid-treatment). The second phase comprising of week-4 to week-6 measures indicates the status in the second half of the treatment phase (end-treatment). The final phase comprises of week-7 to week-10, the washout phase (washout). In order to investigate the change in BFI between the three study arms, we are using a mixed-effects regression analysis with average BFI in the three phases as the outcome, phase as the within-subjects factor, and group (RA, SA, standard of care) as the primary between-subjects factor. The baseline BFI level will be used as a covariate. We shall further adjust the model for key demographic variables such as age and ethnicity, as well as the baseline HADS score. A *subject* random-effect will be used to account for the clustering effect within the three measurements on the same subject. Of primary interest is the group*phase interaction, the significance of which will indicate differences in extent of decrease between the arms. Post-hoc analyses of phase difference within each arm will be carried out multiple comparison adjustment. Model diagnostics will be carried out to confirm the distributional assumptions and appropriate corrective actions (e.g. transformation) will be performed as needed.

Two types of fatigue-related measures will be collected from the Actiwatch-S. The first is a daytime activity count per minute, which is calculated by dividing the total daily activity counts by the total daytime minutes. For each phase as defined above, we shall use the corresponding 21-day (or 28-day) average as appropriate. The second type of measure is obtained from the four self-reported readings of the VAS-F. The daily averages are obtained from the non-missing entries each day, and are then combined to provide the phase average the same way as the daytime activity count. The analytical framework is identical to that for BFI, albeit with the changed outcome.

A secondary analysis with BFI will also be carried out which investigates the difference in proportion of severely fatigued subjects (BFI ≥ 6) across the three study arms. The analytical framework will be that of a mixed-effects logistic regression model with severe BFI (yes/no) being the dichotomous outcome. Apart from group, phase, and group*phase interaction as primary covariates, the model will also be adjusted for baseline status of severity. Remaining covariates in the model are identical to those in the continuous BFI model. The clustering effect due to subject will be accounted for using a generalized estimating equations approach.

In order to assess the extent of association between the improvement in FACT-B and improvement in fatigue measure (specific aim #1 letter D), we shall create the change-scores (week 6 – baseline, week 10 – baseline) for FACT-B (both total and component scores), and BFI. Then we shall fit a linear mixed model similar to that for the original BFI analysis, with BFI change-score as outcome, and the FACT-B change-score as a time-varying covariate. There will not be any phase or week variable in the model. We shall restrict this analysis to the SA and RA arms only so that the group variable will consist of two levels. A group*FACT-B interaction in the model will investigate any differential association across the arms between the change-scores. The additional covariates and clustering adjustment would be similar to the BFI analysis.

Secondary Aims:

1. Assessing difference across study arms with respect to sleep parameters

One of the most widely used sleep measures is the PSQI, which shall be administered at baseline, week 6 and week 10. We shall follow the scoring convention followed for PSQI which constructs scores for seven components, namely sleep quality; sleep latency; sleep duration; habitual sleep efficiency; sleep disturbances; use of sleep-promoting medication; and daytime dysfunction, all derived from the original PSQI. The component scores are summed to obtain a total score, the primary outcome for the proposed study. The modeling framework and subsequent analysis will be similar to that for BFI with the phase variable replaced by a continuous week variable. A logistic regression analysis will be carried out with a dichotomous outcome indicating whether PSQI > 7 or not since the threshold of 7 is considered an indicator for sleep quality in BC survivors. A number of secondary sleep outcomes will be obtained using a conjunction of the Actiwatch-S-S data and sleep diary. These will include measures of total sleep time (minutes spent asleep each night) and sleep efficiency (total sleep time/time in bed* 100). These can all be analyzed using the models and methods described above within the mixed linear or logistic regression modeling framework as appropriate.

2. Assessing differences in time to onset and time to relapse (after treatment ends) across the acupressure arms

Time to onset of the acupressure effect will be investigated by studying the treatment subgroups, namely RA and SA. For fatigue, we shall use the daily average values of VAS-F. Time to onset will be calculated as the first time (in days) the VAS falls down by 3 points in comparison to the baseline. We shall restrict this analyses to subjects whose baseline VAS-F is at least 3. A proportional hazards regression model will be employed to analyze the time to onset data. Treatment arm (RA vs. SA) will be used as the primary covariate in the analysis. Independent variables used in the earlier regression analyses for the primary and secondary aim 1 will be used as additional covariates.

For sleep, we shall use the VAS-SQ measure to carry out our time to onset analysis. As in the case of fatigue, a 3-point drop from baseline in VAS-SQ for the first time will be considered as onset. Since the VAS-SQ measures are weekly, we shall employ a *discrete survival analysis* technique to analyze this data. A discrete survival model is a logistic regression model of the discrete-time hazards, and is easily implemented in most standard statistical softwares. As in the case for VAS-F, this analysis will be confined to subjects with a baseline VAS-SQ score of 3 or more.

We would analyze similarly the time to relapse since the end of treatment, which is defined as the first time a 3-point increase (VAS-F or VAS-SQ) from the six-week value, occurs. This part of the analysis will be restricted to subjects for whom an onset has occurred.

All statistical analyses will be carried out in SAS 9.3 and PASW version 19.

#### Power analysis

Our power analysis is based on our primary aim of comparing fatigue as measured by BFI across the three study arms.

We compute the power via simulation using a mixed effects model with a between-subject factor group (3 levels), a within-subject factor phase (2 levels), group*phase interaction and a random subject effect. The mean BFI values at mid-treatment were assumed to be 4, 3, 3, respectively for standard care, SA and RA arms whereas the means were taken to be 4, 3, 2 at the end-treatment point in the same arms. The between-subject variance is assumed to be 4 at all time-points whereas the variance of the random subject component is taken to be 4 also (yielding an intra-class correlation of 0.5). These assumed values are estimates based on our pilot data. For this configuration, the power for detecting the difference between groups is more than 0.95 and the power for detecting a significant phase*group interaction is 0.82 with a sample size of 100 per treatment arm and a 5% level of significance. In the second model discussed in the analysis section with phase comprising of end-treatment and washout, if we assume the washout point BFI values to be 5, 4, 3, in the standard care, SA, and RA arms, respectively, the power for detecting either the phase effect or group effect are both around 99%.

We also have powered the study to allow us to observe differences in sleep parameters. The extent of overlap between fatigue and sleep disturbances in BC survivors is currently unknown. Research indicates that 51% of BC survivors experience sleep disruptions and 19% meet diagnostic criteria for insomnia [35]. While fatigued women are most likely enriched with individuals experiencing sleep disturbances we will conservatively assume that roughly 51% women will have some significant sleep disturbances and 19% will have insomnia per treatment arm. As such, with 100 women per treatment group we will be overpowered for detecting a difference in fatigue and sufficiently powered to detect changes in key sleep measures. For example, using the PSQI as a basis for powering differences in sleep quality from previous studies the mean PSQI in BC patients ranges from 6.84 ± 0.376 to 7.16 ± 0.325.(60,74) A 1 point decrease in the PSQI from ~7 to ~6 is considered clinically significant in this population [33]. The mean PSQI values at mid-treatment are assumed to be 7, 6, 6, respectively in standard care, SA and RA arms whereas the means are taken to be 7, 6, 5 at the end-treatment point in the same arms. Assuming an intra-subject correlation of 0.5, we have powers above 99% to detect any of the group, phase, and group*phase interaction effects with a sample size of 100 per treatment arm and a 5% level of significance.

All power calculations are carried out on the basis of 100 simulations in the software PASS 2008 (NCSS, Kaysville, Utah, USA).

#### Data safety monitoring plan (DSMP)

The PIs review study progress weekly with study staff, and problems with or pertaining to study subjects are be communicated immediately. The entire research team meets monthly to review progress and any problems encountered. This team includes one physician, a PhD trained nurse and trained acupuncturist who are highly experienced with cancer control trials. The PIs are notified when an AE occurs and determine the attribution and relatedness of each adverse event. All AEs must be given to the PI within 48 hours if involving a death or life threatening event, or within one week, if serious (not-life threatening/death) or non-serious. Lastly, we work with the UM Prevention Research Base DSMB which meets monthly by means of regularly scheduled meetings.

Composition of the UM Prevention Research Base DSMB: the PI is present in an open session portion of the meeting and absent in a closed session. All DSMB official subjects in the review of confidential data and discussions regarding continuance or stoppage of a study have no conflict of interest and no financial stake in the research outcome. The current UM Prevention research base Data and Safety Monitoring Committee is Chaired by the Dr. Mack Ruffin and comprised of Faculty members from the departments of gastroenterology, Family Medicine and Hematology/Oncology. At least 3 faculty members, not including the study PI, must be present to have quorum. If the DSMB cannot meet face-to-face, a conference call is acceptable. Prior to each meeting, the UM Prevention Research Base clinical research associate distributes a standard summary report detailing accrual, new publications or presentations relevant to the ongoing project, quality control audit information, any ethical concerns, patient-subject complaints and adverse events or serious adverse events.

Our study was initially reviewed by The UM Prevention Research Base DSMB beginning in the second year from initial funding and monthly after that. The DSMB also reports its findings of any adverse events or decisions regarding modification of the protocol to the University of Michigan IRB committee.

## Discussion

This clinical trial will help to determine the efficacy and acceptance of a self-administered acupressure technique in BC survivors for PCRF. In order to answer this question, we have developed a protocol and study design with numerous unique aspects. One unique study design is utilizing nurses to teach acupressure to participants. If nurses can successfully learn and teach participants how to administer acupressure it may demonstrate the possibility to teach nurses acupressure in “real world” settings such as Family Medicine practices. Another unique aspect of this study is the use of two sets of acupressure points that are hypothesized to work in an opposing fashion. Two of our pilot studies demonstrated that by using opposing acupoints (stimulating and relaxing) we saw statistically significant differences between treatment groups [13,14]. This effect remains consistent despite using the design in two distinct samples (students and cancer survivors) and while examining different outcomes (fatigue severity and sleepiness). Many previous acupressure studies using various types of sham or control acupressure and or acupuncture find no difference between sham and active treatment, although normally both treatment arms are better than standard of care. This has led some to theorize that acupressure and acupuncture effects are non-specific and should be viewed as placebo only. Others propose that all acupoints or perhaps all places on the body act in identical fashion when stimulated making it unimportant what points are chosen for treatment and rendering the concept of control/sham acupoints meaningless. Researchers struggle with these challenges in an attempt to demonstrate specific effects for various acupuncture treatments. Our research design appears to address this challenge of showing a significant difference between treatment arms and showing a specific effect for a given set of acupoints.

One other unique point of this trial is the deconstruction of the study intervention. In particular, we are examining how long it will take for acupressure to have an effect on a given outcome by collecting weekly, and in the case of fatigue severity, daily information. In the case of fatigue measures we are also observing if acupressure works through steady cumulative changes or through sudden dynamic effects. Moreover, we are able to observe if different sets of acupoints create different patterns. Very few studies have attempted to assess these effects of acupuncture or acupressure treatments. This has led some researchers to posit that negative findings in acupressure and acupuncture studies could be due to false negative results from too few total number of treatments, inappropriate frequency of treatments and inadequate length of the treatments. At least 2 studies support the idea that fewer sessions per week or short length of acupuncture treatment are not as effective at decreasing pain. Harris and colleagues [14] found that 3 acupuncture sessions weekly provided more pain relief than 1 session weekly (p = 0.039). Another research group [36] discovered that the difference between sham and true acupuncture for pain was not evident at 8 weeks but was statistically significant at 14 weeks. Our study will help to clarify issues of how long and the pattern of change needed for change in the study outcomes.

Our protocol also has several distinctive aspects including that cancer survivors are often only seen by their oncologist once a year, or not at all, depending on the number of years from diagnosis. Instead they often obtaining health care from a variety of medical specialist including, but not limited to, family medicine, internal medicine, and gynecology. Cancer survivors are also spread over large geographic areas leading them to seek health care in a wide variety of locations including private clinics, community hospitals and large health systems. These characteristics of cancer survivors are barriers to traditional recruitment methods where patients in a clinic are approached for potential study participation. To overcome these challenges we have incorporated several unique designs into our study. First, to identify and recruit participants we work with the Michigan Tumor Registry. As the tumor registry contains more than 95% of all of the cancer cases in Michigan we are able to bypass clinic recruitment instead opting for mailings to all potential breast cancer survivors within a certain county and date of diagnosis. Also, our protocol makes use of the MSU County Extension offices within several Michigan Counties to conduct study visits. By locating our screening and future study visits in the same county as we are recruiting we minimize travel distances for participants, while maximizing the area from which we can recruit for the study. The MSU County Extension offices provide locations by county throughout all 50 States. As such, they provide one model in which to conduct multi-site studies with populations such as cancer survivors who are utilizing providers in a wide range of specialties or not seeing health care providers.

Along with numerous strengths our study also has several limitations including inability to double blind the study and lack of an objective measure of fatigue. Currently, there are no biomarkers for measuring fatigue. Consequently, fatigue is measured using subjective questionnaires only. While fatigue is a subjective experience, the presence of high amounts of variability between and within participants’ fatigue, over even a short time frame of a day, argue the benefit of pairing objective measures of fatigue with subjective questionnaires. We have included the objective measurement of physical activity and sleep measures using actigraphy. While actigraphy data does not give an objective measure of fatigue it does give insights into the effect of fatigue on daily activity and sleep. Another limitation is our inability to double-blind the participants as to study assignment. Data from our pilot study [13] demonstrated that participants were blinded as to the acupressure treatment to which they were randomized. However, this is not the case between women in this study who are randomized to one of the two acupressure arms and the standard of care arm. Despite this limitation, it is an important study design to be able to compare acupressure treatment to standard of care for fatigue in these women. By having this comparison we will be able to have a preliminary idea about the comparative effectiveness of acupressure compared to standard of care and have a clearer idea of what is the placebo versus the real effect of acupressure treatment.

In summary, this is a protocol examining two opposing types of self-administered acupressure (stimulating and relaxing) compared to standard of care for PCRF in 300 BC survivors. This protocol has many strengths compared to the traditional randomized, double-blind design. In particular, it utilizes local community resources in the form of MSU County Extension Services for study visits, population-based recruiting via the MTR, and nurses to teach the acupressure intervention. This study is scheduled to be completed with recruitment in the Winter of 2014 with published results in 2015.

## Abbreviations

PCRF: Persistent cancer-related fatigue; BC: Breast cancer; RR: Risk ratio; CI: Confidence interval; RA: Relaxation acupressure; SA: Stimulating acupressure; TCM: Traditional Chinese Medicine; RCT: Randomized controlled trials; PSQI: Pittsburgh Sleep Quality Index; MTR: Michigan Tumor Registry; MDCH: State health department; SEER: Surveillance, Epidemiology, and End Results; NAACCR: North American Association of Central Cancer Registries; HADS: Michigan State University (MSU)(Hospital Anxiety and Depression Scale; PSQI: Pittsburgh Sleep Quality Index; VAS: Visual Analog Scale; BFI: Brief fatigue Inventory; BSAPQ: Breast and Surrounding Area Pain Questionnaire; BPI: Brief Pain Inventory; PRIME-MD: Patient Health Questionnaire; LTQL: Long Term Quality of Life; GSE: General Self-Efficacy Scale; Actiwatch-S- S: Actiwatch-S-Score; AE: Adverse events; DSMP: Data Safety Monitoring Plan.

## Competing interests

The authors declare they have no competing interests.

## Authors’ contributions

SMZ and REH designed and initiated the study. SMZ, PML, JTA, SLM, AS, and GKW contributed significantly to the development of the protocol. AS developed the statistical analysis plan. GKW developed the recruitment plan. All authors read and approved the final manuscript.

## Pre-publication history

The pre-publication history for this paper can be accessed here:

http://www.biomedcentral.com/1472-6882/12/132/prepub
